# 1/*f* Noise Modelling and Characterization for CMOS Quanta Image Sensors

**DOI:** 10.3390/s19245459

**Published:** 2019-12-11

**Authors:** Wei Deng, Eric R. Fossum

**Affiliations:** Thayer School of Engineering, Dartmouth College, Hanover, NH 03755, USA; Eric.R.Fossum@dartmouth.edu

**Keywords:** 1/*f* noise, mobility fluctuation, quanta image sensors, QIS, CMOS image sensors, CIS

## Abstract

This work fits the measured in-pixel source-follower noise in a CMOS Quanta Image Sensor (QIS) prototype chip using physics-based 1/*f* noise models, rather than the widely-used fitting model for analog designers. This paper discusses the different origins of 1/*f* noise in QIS devices and includes correlated double sampling (CDS). The modelling results based on the Hooge mobility fluctuation, which uses one adjustable parameter, match the experimental measurements, including the variation in noise from room temperature to –70 °C. This work provides useful information for the implementation of QIS in scientific applications and suggests that even lower read noise is attainable by further cooling and may be applicable to other CMOS analog circuits and CMOS image sensors.

## 1. Introduction

The Quanta Image Sensor (QIS) was proposed in 2005 [1] as a possible next-generation solid-state image sensor after charge-coupled devices (CCDs) and CMOS image sensors (CIS). QIS features high temporal-spatial resolution and has a deep sub-electron read noise that allows photon counting. For accurate photon counting, the QIS read noise target is below 0.15 e^−^ rms [2] although single electron quantization becomes apparent below about 0.45 e^−^ rms. Dartmouth previously reported a megapixel QIS achieving room-temperature single-photon discrimination without avalanche gain with average read noise of 0.21 e^−^ rms [3].

In CCDs, CIS, and CMOS QIS, the signal sense node (the floating diffusion, “FD”) is connected to the gate of a source-follower amplifier. Currently, it is believed that noise from the in-pixel source-follower (SF) transistor dominates the read noise of QIS and high-sensitivity CIS. SF noise is composed of several different types of noise, mainly 1/*f* noise or flicker noise, random telegraph noise (RTN), and thermal noise (or Johnson–Nyquist noise). The origin of RTN is usually attributed to conduction carrier trapping and re-emission at the Si-SiO_2_ interface, while the theory of thermal noise is well-established. Nevertheless, the physical origin of 1/*f* noise has not been well established.

For more than 60 years, 1/*f* noise has been researched. There are three well-known 1/*f* noise models, the McWhorter number fluctuation model [4], the Hooge mobility fluctuation model [5], and the Berkeley unified model [6]. McWhorter’s model considers the 1/*f* noise origin to be the trapping/de-trapping-induced number fluctuation of conduction carriers. Hooge’s model is an empirical model which considers the origin as phonon-scattering-induced mobility fluctuation. Researchers tried to study the physical background of Hooge’s alpha parameter [7], but there is no widely accepted explanation. McWhorter’s model gained popularity after the discovery of RTN [8]. An ensemble of traps with a wide distribution of time constants can yield a 1/*f* noise spectrum. However, as technology nodes scale further, researchers can easily observe the RTN induced by a single trap or several traps. Even for these RTN devices, the background noise after removing the RTN still shows a 1/*f* trend [9]. The Berkeley unified model considers the number fluctuation and the correlated mobility fluctuation as the origin of 1/*f* noise. Different from the mobility fluctuation in Hooge’s model, which is a bulk effect, this model considers the mobility fluctuation induced by scattering from the charge near the Si-SiO_2_ interface.

QIS is designed to be sensitive to single photoelectrons, due to the very low sense node capacitance. When single-trap or multi-traps-induced RTN is present in a QIS device, it can be easily observed even at room temperature, as will be shown later. However, there exists a lower-level of background noise in addition to the RTN. With higher sensitivity and smaller SF gate dimensions, the need for a mobility fluctuation model to explain the measured noise may be stronger than for other image sensor SFs, or perhaps there is more to SF noise than just charge trapping and mobility fluctuation in the SF transistor. This paper discusses different 1/*f* noise models and fits the measured SF 1/*f* noise from a QIS prototype chip using these models instead of the widely-used fitting model [10], which only shows the 1/*f* and area-scaling trend and includes all other effects into a process-dependent coefficient. We find that the mobility fluctuation-based model fits the best. However, as is discussed in the conclusion of the paper, there are a number of reasons why the physical interpretation associated with this model seems inappropriate for our device, and the fit may only mean that the mathematical form fits and other physical interpretations or explanations are needed.

## 2. Models

The three (3) main historical models for 1/*f* noise are now reviewed.

### 2.1. McWhorter Number Fluctuation-Based 1/f Noise Model

The number fluctuation model, originally proposed by McWhorter in 1955 [4] for Germanium surfaces and later applied by others to MOSFETs, considers the 1/*f* noise origin to be the trapping/de-trapping-induced number fluctuation of channel carriers, primarily at the Si-SiO_2_ interface. The normalized power spectrum density (PSD) of 1/*f* noise, due to the number fluctuation, is usually given by [11]

(1) $$\frac{S_{I_{b}}\left(f\right)}{I_{b}^{2}}=\frac{k^{*}}{f}\frac{2}{C_{ox}^{2}WL}\frac{1}{{\left(V_{GS}-V_{th}\right)}^{2}},$$

where *I_b_* is the bias current. $k^{*}=\frac{q^{2}D_{t}\left(E_{F}\right)kT}{\ln\left(\tau_{2}/\tau_{1}\right)}$ is a coefficient related to the tunneling possibility between channel and gate oxide traps. *q* is elementary charge, *D_t_* (*E_F_*) is the active trap density in the vicinity of the Fermi level (*E_F_*), *k* is the Boltzmann constant, *T* denotes temperature, and *τ*_1_ and *τ*_2_ are the lower and upper boundaries of time constants involved in the trapping/de-trapping process. *f* is frequency. *C_ox_* is oxide capacitance per unit area. *W* and *L* are transistor width and length, respectively. *V_GS_* is gate-source voltage of the source follower and *V_th_* is the threshold voltage. Using the simple, long-channel drain-current model in saturation region $I_{b}=\frac{1}{2}\mu C_{ox}\frac{W}{L}{\left(V_{GS}-V_{th}\right)}^{2}$ where *μ* is the nominal mobility (note that for our devices such a model may be only marginally appropriate), (1) can be expressed as

(2) $$S_{I_{b}}\left(f\right)=\frac{q^{2}D_{t}\left(E_{F}\right)kT}{\ln\left(\tau_{2}/\tau_{1}\right)}\frac{\mu I_{b}}{C_{ox}L^{2}}\frac{1}{f}.$$

In the QIS and CIS devices, the output of the SF is connected to a correlated double sampling (CDS) circuit, which is commonly used to reduce the reset noise from the pixel floating diffusion node. SF 1/*f* noise will be filtered by the CDS circuitry, and the transfer function is given by [12]

(3) $$H_{CDS}\left(f\right)=2\;\sin\left(\pi f\Delta t\right),$$

where Δ*t* denotes the time difference between the reset sampling and signal sampling. The dominant time constant due to the SF transconductance *g_m_* is denoted by *τ*_D_ = *C_col_*/*g_m_*, where *C_col_* is the column capacitance. The bandwidth of the SF introduces a low-pass filter and the cutoff frequency is given by *f*_c_ = 1/(2π*τ_D_*). The transfer function (assuming the gain is 1) can be written as

(4) $$H_{LP}\left(f\right)=\sqrt{\frac{1}{1+{\left(f/f_{c}\right)}^{2}}}.$$

A programmable gain amplifier (PGA) with a switchable analog gain *G_A_* is implemented to amplify the signal and suppress the noise contribution from the subsequent signal readout electronics. The noise will also be amplified by the PGA before it is digitized by the off-chip ADC. Assuming additional PGA and ADC noise is negligible, the SF 1/*f* noise power, due to the bandpass filtering and amplification, is thus given by

(5) $$n_{1/f}^{2}=\int_{0}^{\infty }\frac{S_{I_{b}}\left(f\right)}{g_{m}^{2}}H_{LP}^{2}\left(f\right)H_{CDS}^{2}\left(f\right)G_{A}^{2}df.$$

Substituting Equations (2)–(4), into Equation (5), the SF 1/*f* voltage noise, due to the number fluctuation can be written as

(6) $$n_{1/f}=\eta_{M}G_{A}\sqrt{\frac{kT\Psi \left(\pi f_{c}\Delta t\right)}{WL}},$$

where $\eta_{M}$ is defined as $\eta_{M}=\frac{q}{C_{ox}}\sqrt{\frac{2D_{t}\left(E_{F}\right)}{\ln\left(\tau_{2}/\tau_{1}\right)}}$, and $\Psi \left(\pi f_{c}\Delta t\right)=\int_{0}^{\infty }\frac{\sin^{2}\left(\pi f_{c}\Delta tx\right)}{x\left(1+x^{2}\right)}dx$ is a function of the cutoff frequency *f*_c_ and CDS ∆*t* [13], while *x* represents *f*/*f*_c_.

### 2.2. Hooge Mobility Fluctuation-Based 1/f Noise Model

Hooge’s model, introduced by Hooge in 1969 [5,14], considers the origin of 1/*f* noise to be bulk-related mobility fluctuation. We note that Hooge was considering relatively low electric fields in his modelling. Mobility, which itself is an average value giving an average or expected net carrier velocity in response to an electric field, has a variance or fluctuation that Hooge related to Brownian-motion effects but could also be related to variances in scattering. In a normal image sensor operation, the SF is biased in saturation. The normalized PSD of 1/*f* noise of a SF biased in the saturation region, due to mobility fluctuation, is given by [11]

(7) $$\frac{S_{I_{b}}\left(f\right)}{I_{b}^{2}}=\frac{\alpha_{H}}{f}\frac{2q}{C_{ox}WL}\frac{1}{\left(V_{GS}-V_{th}\right)},$$

where *α_H_* is Hooge’s parameter. Using the drain current model in the saturation region (albeit a high electric field), the PSD can be written as

(8) $$S_{I_{b}}\left(f\right)=\frac{\alpha_{H}}{f}\frac{2^{3/2}q\mu^{1/2}I_{b}^{3/2}}{C_{ox}^{1/2}W^{1/2}L^{3/2}}.$$

Substituting Equations (3), (4) and (8), into Equation (5), the SF 1/*f* voltage noise, due to the Hooge mobility fluctuation, can be written as

(9) $$n_{1/f}=\eta_{H}G_{A}\frac{I_{b}^{1/4}\Psi {\left(\pi f_{c}\Delta t\right)}^{1/2}}{W^{3/4}L^{1/4}},$$

where *η_H_* is a mobility-dependent coefficient and is defined as $\eta_{H}=\frac{2^{5/4}q^{1/2}\alpha_{H}^{1/2}}{\mu^{1/4}C_{ox}^{3/4}}$.

### 2.3. Modified Berkeley 1/f Noise Model

The Berkeley unified model, originally proposed by Hung et al. in 1990 [6], considers both the carrier number fluctuation and the correlated surface mobility fluctuation. Since the number fluctuation is usually induced by traps and there are few interface traps in the majority of QIS devices discussed, number fluctuation, due to traps, is not likely the origin of 1/*f* noise. In our modified Berkeley model, only the contribution from the scattering-induced mobility fluctuation is considered [15]. The scattering is due to the trapped charge or surface charge near the Si-SiO_2_ surface. The PSD of 1/*f* noise due to charge-scattering-induced mobility fluctuation is given by [6]

(10) $$S_{I_{b}}\left(f\right)=\frac{kTI_{b}^{2}}{\gamma fWL^{2}}\int_{0}^{L}N_{t}\left(E_{fn}\right)\alpha^{2}\mu^{2}dx,$$

where γ is the attenuation coefficient of the electron wave function in the oxide, typically 10^8^ cm^−1^ for Si-SiO_2_ system. *N_t_* (*E_fn_*) is trap density at the quasi-Fermi level *E_fn_*, and α denotes the scattering coefficient. Assuming *N_t_* (*E_fn_*) is uniform in space [6], the PSD can be simplified as

(11) $$S_{I_{b}}\left(f\right)=\frac{kTI_{b}^{2}\alpha^{2}\mu^{2}N_{t}}{\gamma fWL}.$$

Substituting Equations (3), (4) and (11), into Equation (5), the SF 1/*f* voltage noise can be written as [15]

(12) $$n_{1/f}=\eta_{B}G_{A}\frac{\sqrt{kTI_{b}\Psi \left(\pi f_{c}\Delta t\right)}}{W},$$

where *η_B_* is a mobility-dependent coefficient and is defined as $\eta_{B}=\sqrt{\frac{2\alpha^{2}\mu N_{t}}{\gamma C_{ox}}}$.

## 3. Experimental Setup

**Figure 1:**
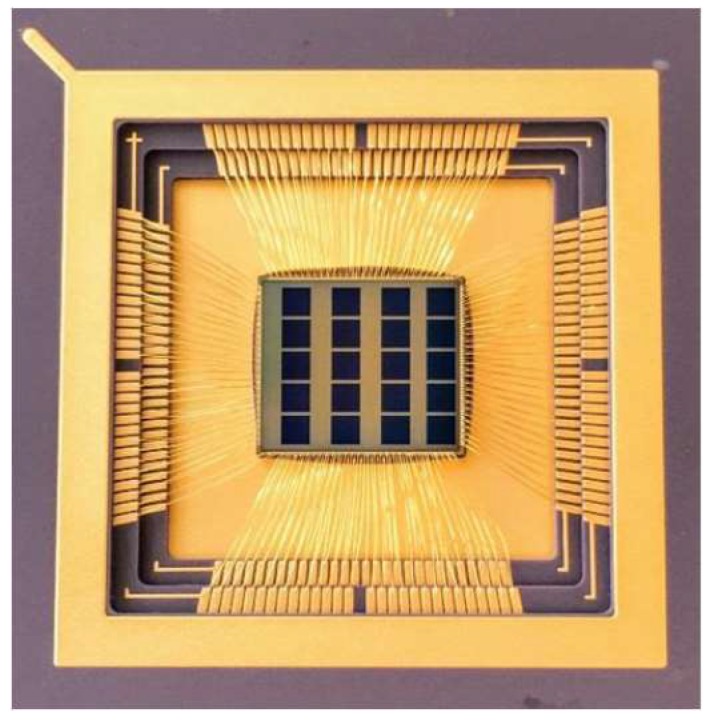
20 × 1Mjot QIS test chip.

**Figure 2:**
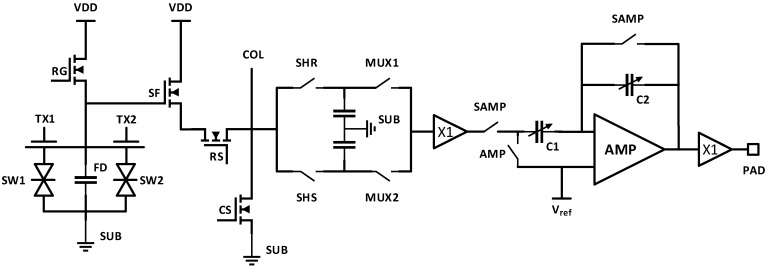
Schematic of the readout signal chain for analog output.

A QIS prototype chip fabricated by TSMC in a modified 45/65 nm stacked backside-illuminated (BSI) CIS process [3] is shown in Figure 1, which contains twenty different 1Mjot QIS arrays. (A jot is a specialized pixel with a low full-well capacity and high conversion gain.) Figure 2 depicts the schematic of the readout chain. A two-way shared readout is implemented in the devices. The incident photons are absorbed by the photodiode and generate corresponding photoelectrons. The storage well (SW) collects the photoelectrons. During the charge transfer, the photoelectrons will be transferred to the floating diffusion (FD), thereby changing its potential, which is then read out by the in-jot SF. The jot output from the pixel wafer will be sent to the ASIC wafer through inter-die connections. A CDS circuit with two storage capacitors is implemented after the SF to process the jot signal. The samples in the CDS units are selected by a multiplexer and connected to a unity-gain buffer. The output of the unity-gain buffer is then connected to a PGA, where the signal is amplified with switchable analog gain that ranges from 2 V/V to 40 V/V. The output of the PGA is then sent to another unity-gain buffer, which drives the output pads so the signal can be readout off-chip. An off-chip 14-bit ADC is used to quantize the signal.

**Figure 3:**
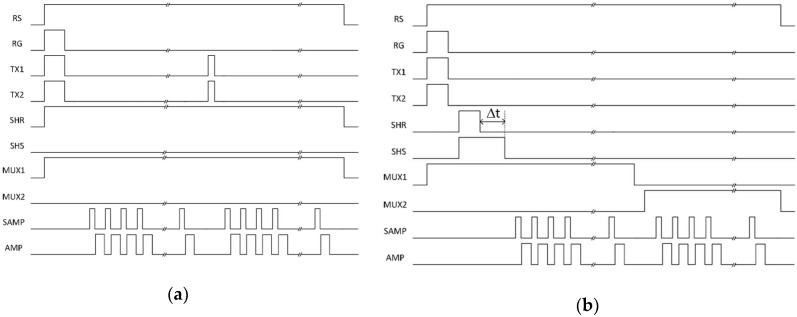
Timing diagram used for (**a**) photon-counting histogram testing; (**b**) SF noise testing.

The operation timing diagram is illustrated in Figure 3. The sensor is read out using a rolling shutter like conventional CMOS image sensors. After a row is selected, the jots are reset. The reset level is read out through the signal chain of Figure 2. However, in the reported measurements, only one branch of the CDS unit was used with the SHR and MUX1 switches always closed for photon counting histogram (PCH) testing. Figure 3a shows a timing diagram depicting this testing methodology. The PGA is set to a gain of 10×. The reset level is amplified and sampled by the off-chip ADC 42 times at 667 kHz. After the TX gates have been pulsed, the new signal level is amplified and sampled by the ADC another 42 times. The reset level is determined by taking the mean of 38 of the 42 samples taken by the ADC for the reset level and the signal level is calculated similarly. CDS is performed digitally by subtracting these two values.

Figure 3b illustrates the timing diagram of another testing methodology for SF noise testing, where the filtering technique for noise suppression is applied. At the beginning of the measurement, the FD was reset and the SWs of the pixel were emptied. The SF output was sampled by two CDS capacitors sequentially. Adjusting the ∆t between the falling edge of the two CDS sampling signals, the SF noise in different frequency regions can be characterized. The CDS samples were read out by the PGA using a correlated multiple sampling (CMS) process to reduce the noise contributions from the PGA and the subsequent readout chain electronics. With 38 CMS readout cycles, the noise contribution from the PGA and the subsequent readout chain becomes negligible and more CMS cycles do not further reduce the noise. During the testing, the QIS chip was placed in a dark temperature-controlled chamber. Without an optical signal, the total measured noise should only include dark signal noise (negligible due to the very low dark current of 0.07 e^−^/s average at room temperature), the SF noise and the kTC noise from the CDS storage capacitors. The read noise with different types of SFs is investigated at different CDS ∆*t*, bias current, and temperatures. Each temperature testing starts after a certain waiting time (e.g., 3.5 h) to ensure the chip reaches the chamber temperature, which was confirmed by measurement using a thermometer. Future QIS chips with on-chip temperature sensors for more accurate temperature characterization are currently in fabrication.

Devices are tested using photon-counting histogram methodology [16] first, operating in a way depicted in Figure 3a. The noise for 4 types of SFs as shown in Table 1 was measured, two buried-channel MOSFETs of different channel widths and two surface-channel MOSFETs of different channel widths (designated BC014, BC018, SC014, and SC020, where BC/SC denotes buried/surface channel and the number is the channel width, e.g., 014 means 0.14 μm). Each device utilized the tapered reset gate to reduce sense node capacitance [17]. The channel length is 0.27 μm for all devices and a total of 1280 devices of each type were measured.

## 4. Results

### 4.1. Photon Counting Histogram Testing

**Figure 4:**
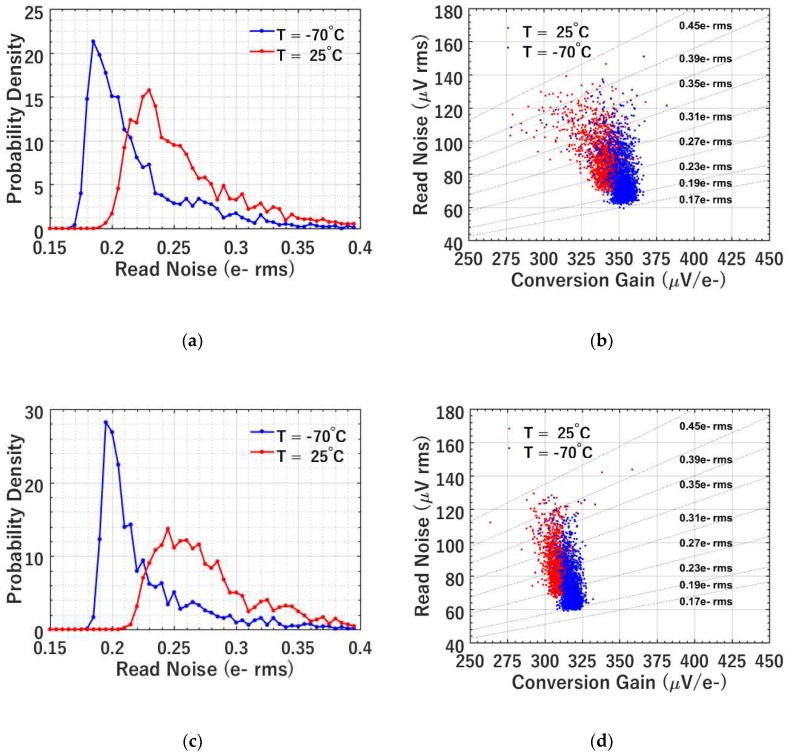
(**a**) A histogram of the read noise of the jots (256 × 8) at −70 and 25 °C with BC014 SF; (**b**) a scatter plot of the voltage-referred read noise versus conversion gain of jots at −70 and 25 °C with BC014 SF; (a small fraction of data with noise higher than 0.45 e^−^ rms is not shown. Same for (**d**).) (**c**) a histogram of the read noise of the jots (256 × 8) at −70 and 25 °C with BC018 SF; (**d**) a scatter plot of the voltage-referred read noise versus conversion gain of jots at −70 and 25 °C with BC018 SF.

**Figure 5:**
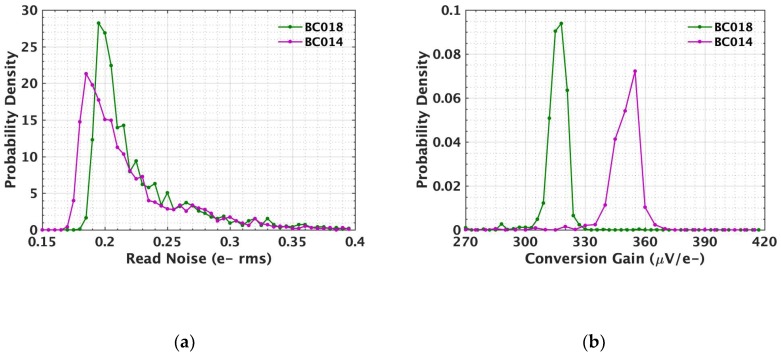
(**a**) A histogram of the read noise of the jots (256 × 8) with BC018 SF and BC014 SF at −70 °C; (**b**) a histogram of the conversion gain of the jots with BC018 SF and BC014 SF at −70 °C.

Figure 4 and Figure 5 show the results from the photon-counting histogram (PCH) testing using the timing diagram of Figure 3a. The PCH of each jot (actually two binned jots which share the readout) was created by repeatedly integrating and reading out the jot for 20,000 samples. The read noise and conversion gain (CG) were then extracted using the valley-to-peak modulation (VPM) method [16]. Figure 4 shows the read noise histograms for jots with BC014 SF and BC018 SF at room temperature and −70 °C. The noise is notably smaller at −70 °C, as shown in Figure 4a. The jots with BC014 SF show an average read noise of 0.26 e^−^ rms with a best-case read noise of 0.19 e^−^ rms at 25 °C, while the average read noise is 0.22 e^−^ rms with a best-case of 0.17 e^−^ rms at −70 °C. The reduction of the read noise at low temperature is mainly due to the reduction of 1/*f* noise [6]. As depicted in Figure 4b, a scatter plot of the voltage-referred read noise versus CG at 25 °C and −70 °C is presented. Dash lines are used to mark the noise in electrons, from which the minimum noise 0.17 e^−^ rms at −70 °C is clearly observed. The CG is higher at low temperature, i.e., 352 μV/e^−^ on average at −70 °C versus 340 μV/e^−^ at 25 °C, likely due to the increase of SF gain as a result of mobility increase thus transconductance increase [18]. The increase of SF gain will reduce the Miller capacitance at the FD node due to gate-source overlap, hence the measured CG is higher at −70 °C. However, the read noise reduction at lower temperatures is not only due to increasing CG, but also SF noise reduction, since CG increases ~4% but input-referred read noise decreases ~15%. Figure 4c,d show the experimental data for jots with BC018 SF. Similar read noise scaling trend with temperature is observed.

**Figure 6:**
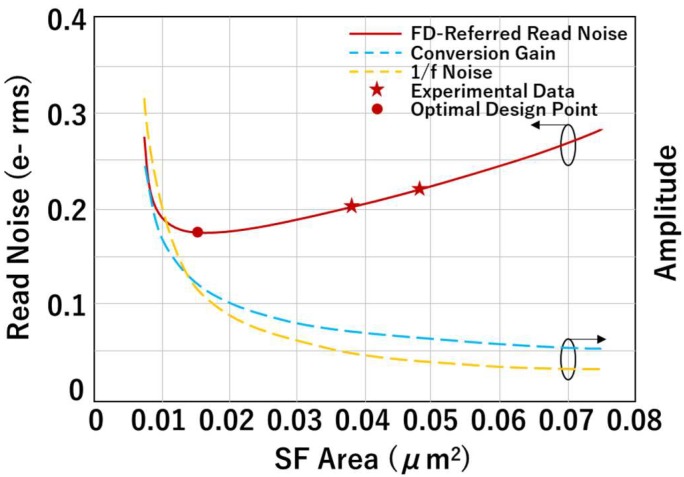
Projected scaling of noise and conversion gain with source-follower area for BC-variety devices in the utilized process.

A comparison is made between the BC014 SF and BC018 SF at −70 °C, as illustrated in Figure 5. Figure 5a shows that BC014 SF has lower noise than BC018 SF. Although BC014 SF has a smaller gate area thus higher 1/*f* noise [6], its CG is higher compared to BC018 SF due to the smaller gate capacitance as shown in Figure 5b. The high conversion gain factor leads to lower FD-referred noise in the end. Figure 6 shows this tradeoff between 1/*f* noise and CG with SF area scaling. The FD-referred 1/f noise can be written as

(13) $$u_{1/f}=\frac{n_{1/f}}{CG},$$

The conversion gain at jot output is given by $CG=\frac{qG_{SF}}{C_{SF}+C_{0}}$, while G_SF_ is the SF gain, $C_{SF}=\beta C_{ox}WL$ is the contribution to FD capacitance from SF gate (β is the equivalent SF gate capacitance coefficient) and *C*_0_ is the contribution from other sources. As the SF area decreases, the CG will keep increasing, due to reduced SF gate capacitance while 1/*f* noise *n*_1/*f*_ also increases. An optimum design point, as marked in Figure 6, should exist and help achieve the minimum FD-referred read noise. Future QIS chips exploring the optimum design point and containing even smaller SF gate area are currently in fabrication.

### 4.2. SF 1/f Noise Testing

**Figure 7:**
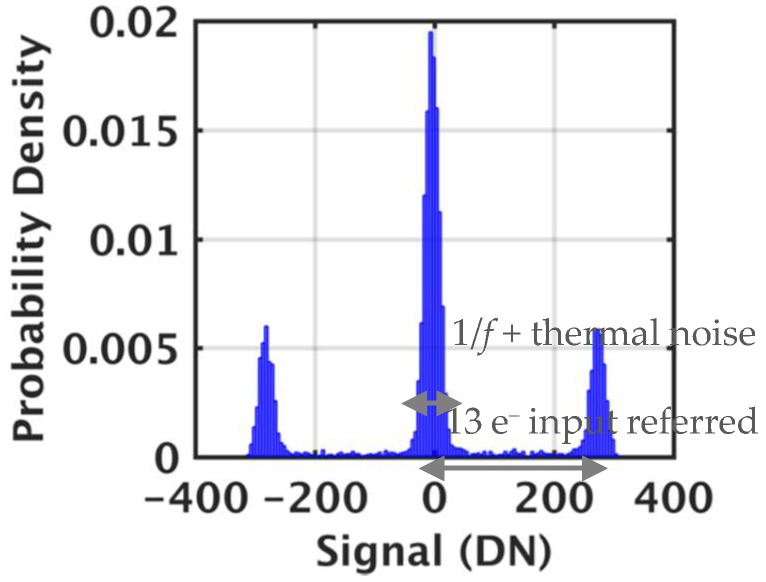
Example of an output signal with RTN (1 DN = 17 μV).

1/*f* noise is measured by applying bandpass filtering using the timing diagram in Figure 3b. The jot output was sampled by two CDS capacitors (550fF) sequentially. The noise in different frequency regions is measured by adjusting CDS Δ*t* [19,20,21]. Figure 7 shows an example of the jot output signal with RTN using this testing methodology. Only about 2% of the devices measured show RTN. Generally, RTN is a large signal. For example, as shown in Figure 7, the input-referred RTN amplitude, after dividing by CG, is 13 e^−^, which is gigantic for single-photon counting. Other devices showed different RTN amplitudes, likely reflecting the location of the trap. For the three peaks of Figure 7, the signal is still spread by residual 1/*f* noise and some thermal noise. For the other 98% of the devices, the measured 1/*f* noise is probably not related to RTN due to lack of this multi-peak RTN signature.

For each type of SF, an array of 256 × 5 jots was tested under different CDS time differences, bias currents, and temperatures. The total measured noise is mainly composed of SF noise and the kTC noise from the CDS capacitors. In 1/f noise analysis, only the quietest 1000 of 1280 measured jots were analyzed for each type of SF [22], thus excluding jots with large RTN and other noisy outliers. (Selecting 1000 jots was a somewhat arbitrary but convenient cutoff.) The kTC noise from the CDS capacitor, which is approximately equal to the measured noise when CDS Δ*t* is 0, was subtracted in the analysis. The obtained SF noise is mainly 1/*f* noise since the SF thermal noise is much smaller after filtering. To identify the origin of the 1/*f* noise, all three 1/*f* noise models introduced before are examined and compared to the experimental results.

#### 4.2.1. 1/*f* Noise versus CDS Δ*t*

**Figure 8:**
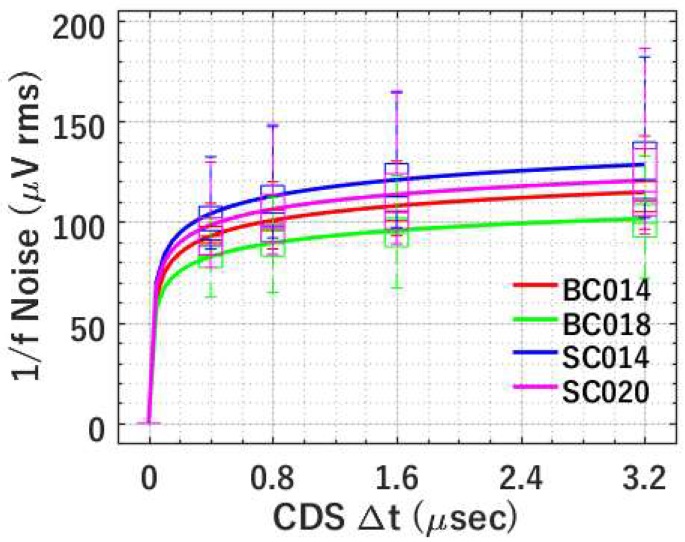
Modelled (using average noise) and measured 1/*f* noise versus CDS Δ*t* at temperature *T* = −70 °C and bias current *I_b_* = 1 μA for 4 types of SFs. The measured data is shown using box plots and the simulated data is shown by solid lines.

According to previously introduced models, 1/*f* noise is dependent on CDS Δ*t*. Figure 8 shows the comparison between the modelled and experimental data for four types of SFs. The measured noise is presented using boxplots, while the modelled noise trend, based on the measured average noise, is shown using solid curves. As shown in Figure 8, the modelling of 1/*f* noise versus CDS Δ*t* characteristics matches the experimental data excellently. 1/*f* noise can be effectively suppressed by decreasing CDS ∆t. Specifically, 1/*f* noise at five different CDS ∆*t*, i.e., 0, 0.4, 0.8, 1.6, and 3.2 μs was measured. All four types of SFs show decreasing 1/*f* noise at smaller CDS ∆*t*, which is consistent with [19,20].

The modelling results shown in Figure 8 can be obtained from any of the three models, i.e., the McWhorter number fluctuation-based model, the Hooge mobility fluctuation-based model, and the modified Berkeley model, but with different *η*. All models fit the experimental data equally well, demonstrating the effectiveness of 1/*f* noise suppression by CDS. On the basis of Equations (6), (9), and (12) *η* from different models can be extracted for each type of SF. Table 2 shows an example of fitted *η* corresponding to Figure 8 with 1 μA bias current at −70 °C. The range of the extracted values of *η* for all 4 types of SFs is pretty tight.

#### 4.2.2. 1/*f* Noise versus Bias Current

1/*f* noise is also characterized at different bias currents. According to Equations (6), (9), and (12), both the Hooge mobility fluctuation-based model and the modified Berkeley model predict a lower 1/*f* noise at lower bias current, while the McWhorter number fluctuation-based model has no bias current dependence.

**Figure 9:**
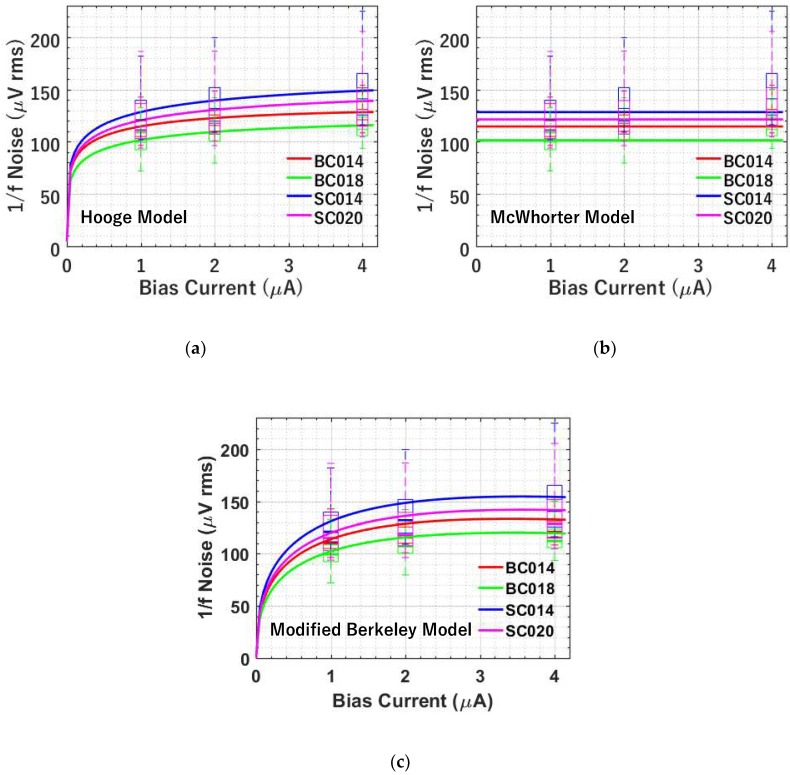
Modelled (using average noise) and measured 1/*f* noise versus bias current at temperature *T* = −70 °C and CDS Δ*t* = 3.2 μs for 4 types of SFs. (**a**) Hooge mobility fluctuation-based 1/f noise model (After fitting using $\eta =\eta_{0}+\eta_{1}lnI_{b}$) versus experiment; (**b**) McWhorter number fluctuation-based 1/*f* noise model versus experiment; (**c**) modified Berkeley 1/*f* noise model (After fitting using $\eta =\eta_{0}+\eta_{1}lnI_{b}$) versus experiment. The measured data is shown using box plots and the simulated data is shown by solid lines.

As shown in Figure 9a, a decreasing trend for 1/*f* noise with decreasing bias current is observed in measurement. However, the modelling result from the Hooge mobility fluctuation-based model shows a stronger dependence on bias current than the measurement. Since *η* includes the scattering effect, it is expected to be dependent on carrier density and therefore the bias current. The higher the bias current, the bigger the carrier density, thus the reduced scattering. Following Equation (16) of Reference [6], but using the bias current instead of electron density, an empirical expression for η is $\eta =\eta_{0}+\eta_{1}lnI_{b}.\;$ The solid line shown in Figure 9a is the best fit with the empirical expression where η_0_ corresponds to the η at 1uA and η_1_ is the fitting coefficient (e.g., −6.2 for BC014, BC018 and SC014, and −7.6 for SC020 in this case). After this bias current dependency fitting for η, the modelling and experimental data match very well.

As depicted in Figure 9b, the modelling result of 1/*f* noise from the McWhorter number fluctuation-based model shows no bias current dependence, while the experimental data indicates the reduced noise at a lower bias current. This discrepancy indicates that the McWhorter number fluctuation-based model has difficulty explaining the observed bias current dependency. Figure 9c illustrates the comparison between the modelling using the modified Berkeley model and the experimental data. Similar to the Hooge mobility fluctuation-based model, the modelling result of 1/*f* noise shows a stronger dependence on the bias current than the measurement. An empirical expression $\eta =\eta_{0}+\eta_{1}lnI_{b}$ is needed to fit this bias current dependence as shown by the solid line. Figure 9c shows a reasonable match after fitting.

Overall, none of the models can be readily fitted with the experimental data in the bias current testing. Both the Hooge mobility fluctuation-based model and the modified Berkeley model show a stronger bias current dependence than the measurement does. However, after introducing the log fitting model, the modelling can fit the experimental data reasonably well as depicted in Figure 9a,c.

#### 4.2.3. 1/f Noise versus SF Size

**Figure 10:**
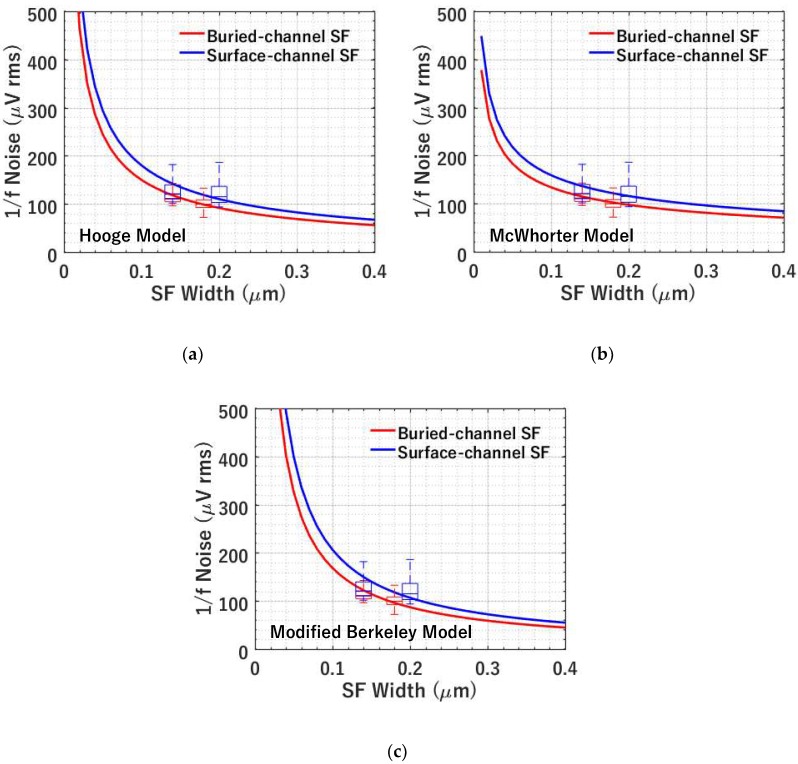
Modelled (using average noise) and measured 1/*f* noise versus SF transistor width at temperature *T* = −70 °C, *I_b_* = 1 μA, and CDS Δ*t* = 3.2 μs for 4 types of SFs. (**a**) Hooge mobility fluctuation-based 1/*f* noise model versus experiment; (**b**) McWhorter number fluctuation-based 1/*f* noise model versus experiment; (**c**) modified Berkeley 1/*f* noise model versus experiment. The measured data is shown using box plots and the simulated data is shown by solid lines.

Figure 10 depicts the noise trend with the SF dimension for different types of conduction channels, i.e., buried channel and surface channel. The average η of BC014 and BC018 is used for modelling the 1/*f* noise versus SF width characteristics for buried-channel SF. Similarly, the average η of SC014 and SC020 is used for 1/*f* noise modelling of surface-channel SF. As shown in Figure 10a, the modelling using the Hooge mobility fluctuation-based model matches the experimental data reasonably. 1/*f* noise is higher for smaller devices, which agrees with previous findings [6]. Buried-channel SFs have lower noise than surface-channel SFs, which is likely due to less scattering.

Figure 10b,c shows the modelling results from the McWhorter number fluctuation-based model and the modified Berkeley model. All models seem to match the experimental data. To better discriminate between 1/*W*^2/4^, 1/*W*^3/4^, and 1/*W*^4/4^ dependencies, devices with more size variations are needed, which are currently in fabrication.

#### 4.2.4. 1/*f* Noise versus Temperature

**Figure 11:**
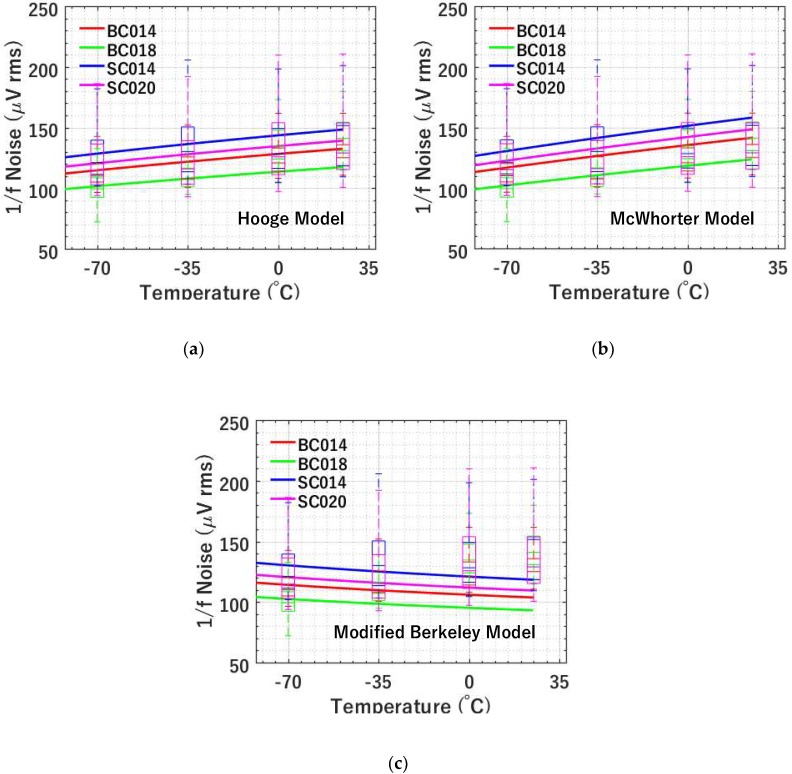
Modelled (using average noise) and measured 1/*f* noise versus temperature for 4 types of SFs at *I_b_* = 1 μA and CDS Δ*t* = 3.2 μs for 4 types of SFs. (**a**) Hooge mobility fluctuation-based 1/*f* noise model versus experiment; (**b**) McWhorter number fluctuation-based 1/*f* noise model versus experiment; (**c**) modified Berkeley 1/*f* noise model versus experiment. The measured data is shown using box plots and the simulated data is shown by solid lines.

As shown in Figure 11, 1/*f* noise is characterized at four different temperatures, i.e., −70, −35, 0, and 25 °C. A decreasing trend with temperature is observed for the measured 1/*f* noise. Figure 11a shows the modelling using the Hooge mobility fluctuation-based model. According to Equation (9), 1/*f* noise is proportional to *η*, while the relationship between η and mobility μ can be written as $\eta \propto \mu^{-1/4}$. In the temperature range of interest, the mobility μ has temperature dependence, i.e., $\mu \propto T^{-3/2}$ [23]. Hence, the temperature dependence of 1/*f* noise is $n_{1/f}\propto T^{3/8}$. Considering this temperature dependence, Equation (9) can be re-written as $n_{1/f}=\eta_{H0}\frac{T^{3/8}}{T_{0}^{3/8}}G_{A}\frac{I_{b}^{1/4}\Psi {\left(\pi f_{c}\Delta t\right)}^{1/2}}{W^{3/4}L^{1/4}}$, where η_H0_ is the *η* at *T*_0_., e.g., *η_H_* from Table 2 at *T*_0_ = −70 °C. As shown in Figure 11a, the solid curves are the modelling results. The modelling and experimental data match excellently. 1/*f* noise is expected to be even lower at temperatures below −70 °C. However, since the current temperature test chamber can only reach as low as −70 °C, 1/*f* noise at lower temperatures is not characterized. Future testing may be conducted at even lower temperature or even cryogenic temperature range.

Figure 11b illustrates the modelling results using the McWhorter number fluctuation-based 1/*f* noise model. However, the modelling shows a stronger temperature dependence than the experimental data does. Comparing the previous Hooge mobility fluctuation-based model to the McWhorter number fluctuation-based model, although both models show decreasing 1/*f* noise at lower temperatures, the former shows a better fit on the temperature dependence.

Figure 11c shows the modelling results from the modified Berkeley 1/*f* noise model. According to Equation (12), 1/*f* noise is proportional to η, while the relationship between η and mobility μ can be written as $\eta \propto \mu^{1/2}$ and the mobility μ has temperature dependence, i.e., $\mu \propto T^{-3/2}$. Hence, the temperature dependence of 1/*f* noise can be written as $n_{1/f}\propto T^{-1/4}$, which indicates that 1/*f* noise increases at lower temperatures. As shown in Figure 11c, the solid curves are the modelling results after considering the temperature dependence of the mobility, which predicts an opposite trend compared to the measurement. The discrepancy between modelling and experimental data indicates that the modified Berkeley model after taking the temperature dependence of mobility into account has problems explaining the measured temperature dependence.

## 5. Discussion

Overall, the Hooge mobility fluctuation-based 1/*f* noise model fits the experimental data better than the other two models. A comparison between different models is shown in Table 3. Note that the Hooge mobility fluctuation-based model shows a $T^{3/8}$ temperature dependence, which fits the experimental data of low-temperature testing the best compared to others. The goodness of fit is also presented in Table 4. All three models can describe the 1/*f* noise versus CDS ∆t characteristics and the noise scaling trend with the device dimension. However, the McWhorter number fluctuation-based model has no bias current dependence and thus has a poor fit, while the modified Berkeley model after considering the temperature dependence of mobility fits the measured temperature data poorly due to a reverse trend. Although none of the models fits the bias current dependence well, the Hooge mobility fluctuation-based model and modified Berkeley model match the experimental data after log fitting [6]. Comparing the three models, Hooge mobility fluctuation model seems to provide a good fit for all parameters under investigation in contrast to earlier modelling results we reported that favored the modified Berkeley model [15]. Meanwhile, since there are almost no traps which lead to number fluctuation in SF and the trap-induced RTN is huge, the mobility fluctuation explanation makes more sense for QIS devices than number fluctuation.

Although the Hooge mobility fluctuation-based 1/*f* noise model fits the best, there are still some conceptual problems with the mobility fluctuation model. Essentially, mobility fluctuation causes transit-time or velocity variation of the carriers leading to the fluctuation in the instantaneous current. Since 1/*f* noise PSD extends into the deep low-frequency region, there should be some corresponding long-time-scale events that lead to the low-frequency noise. However, the time scale of transit-times is usually in pico or nano seconds. Therefore, the mechanism that gives rise to long-time-scale fluctuations is still a mystery. Another outstanding issue is how the transit time fluctuation affects the SF source voltage. When the carriers enter the source, they don’t yet see transit time fluctuation. How does the drain current fluctuation feed back and affect the source voltage? Furthermore, how does a floating SF gate, which is different from a constantly-biased gate, impact the SF noise characteristics? Additionally, what causes the sharp cutoff of the noise histogram at the lower noise end, as shown in Figure 4a, almost as if another limiting noise mechanism is present? All these issues seem hard to explain using the models we have discussed. To address these issues, a new physics-based model or model that more fully includes other circuit elements may be needed. We might conjecture that a more likely origin of source-follower noise in our devices is the fluctuation in emission rate of carriers (electrons) from the source into the very short channel and thus calming this emission process down may be a method for reducing the SF noise. Future work is needed to mathematically model this process and its dependencies on the bias current, gate dimensions, and temperature, especially as emission-induced noise is often considered to be modeled best as shot noise.

Future measurement will be performed on QIS devices to identify whether 1/*f* noise is a bulk effect, i.e., mobility fluctuation, or a surface effect, i.e., number fluctuation or corresponding correlated mobility fluctuation [6]. QIS with a buried-channel JFET (i.e., Junction gate field-effect transistor) SF, which has no gate oxide layer, will be a great candidate to illuminate the 1/*f* noise origin. The measurement results from the QIS with a JFET SF will shine some light on future modelling. In fact, a QIS device with a JFET source-follower has been designed, fabricated, and measured [22,24]. However, possibly due to implant conditions, the performance was very variable between devices of the same type and between the buried-gate and surface-gate JFET devices. In the “good” devices, substantial readout noise was still present, despite our expectation of lower noise. The conversion gain of these devices was higher than MOSFETs, as was predicted by earlier modelling, but not sufficient to overcome residual FET noise. Such residual noise may be more in line with an emission or mobility fluctuation model than a number fluctuation model but improved devices need to be fabricated and measured. Such devices are currently in fabrication.

## 6. Conclusions

In this paper, 1/*f* noise models based on different origins are introduced and compared. Mobility fluctuation makes more sense in explaining 1/*f* noise in QIS devices compared to number fluctuation. Both modelled and measured 1/*f* noise are presented. The Hooge mobility fluctuation-based modelling reasonably matches the measurements, including temperature dependence, but are unsatisfactory when considering transport in our short-channel, low-bias current, high-channel-field devices. The trends nevertheless provide guidance for future cooled-temperature applications. This work suggests possible ways, either by bandpass filtering, SF sizing, or further cooling, to achieve even lower read noise and indicates possible QIS design and operation improvements for scientific applications.

## Figures and Tables

**Table 1:** List of tested devices.

SF Name	BC014	BC018	SC014	SC020
Channel Type	Buried-channel (μm)	Buried-channel (μm)	Surface-channel (μm)	Surface-channel (μm)
*W*/*L*	0.14 /0.27	0.18/0.27	0.14/0.27	0.20/0.27

**Table 2:** Extracted *η* from Figure 8.

SF Type	BC014	BC018	SC014	SC020
*η*_M_	2.55E7	2.50E7	2.85E7	3.15E7
*η_H_*	34.3	36.3	38.4	46.4
*η*_B_	18.4	21.0	21.0	27.3

**Table 3:** Comparison of 1/*f* noise models.

	Mobility Fluctuation Based Model	Number Fluctuation Based Model	Modified Berkeley Model
1/*f* Noise Model	$n_{1/f}=\eta G_{A}\frac{I_{b}^{1/4}\Psi {\left(\pi f_{c}\Delta t\right)}^{1/2}}{W^{3/4}L^{1/4}}$	$n_{1/f}=\eta G_{A}\sqrt{\frac{kT\Psi \left(\pi f_{c}\Delta t\right)}{WL}}$	$n_{1/f}=\eta G_{A}\frac{\sqrt{kTI_{b}\Psi \left(\pi f_{c}\Delta t\right)}}{W}$
Relation with *I_b_* & *T*	$n_{1/f}\propto \eta \left(I_{b}\right)I_{b}^{1/4}T^{3/8}$ ^1^	$n_{1/f}\propto T^{1/2}$	$n_{1/f}\propto \eta \left(I_{b}\right)I_{b}^{1/2}T^{-1/4}$ ^2^

^1,2^$\eta \;\left(I_{b}\right)$ is empirically expressed as $\eta \;\left(I_{b}\right)=\eta_{0}+\eta_{1}lnI_{b}$.

**Table 4:** Qualitative fit between modelling and experiment.

	Mobility Fluctuation Based Model	Number Fluctuation Based Model	Modified Berkeley Model
1/*f* Noise vs. CDS ∆*t*	Excellent	Excellent	Excellent
1/*f* Noise vs. *I_b_*	Good^1^	Poor	Good^2^
1/*f* Noise vs. SF Width	Good	Good	Good
1/*f* Noise vs. *T*	Excellent	Good	Poor

^1,2^ After fitting using $\eta =\eta_{0}+\eta_{1}lnI_{b}$.
